# Cerebellar ataxia, neuropathy, vestibular areflexia syndrome due to RFC1 repeat expansion

**DOI:** 10.1093/brain/awz418

**Published:** 2020-02-10

**Authors:** Andrea Cortese, Stefano Tozza, Wai Yan Yau, Salvatore Rossi, Sarah J Beecroft, Zane Jaunmuktane, Zoe Dyer, Gianina Ravenscroft, Phillipa J Lamont, Stuart Mossman, Andrew Chancellor, Thierry Maisonobe, Yann Pereon, Cecile Cauquil, Silvia Colnaghi, Giulia Mallucci, Riccardo Curro, Pedro J Tomaselli, Gilbert Thomas-Black, Roisin Sullivan, Stephanie Efthymiou, Alexander M Rossor, Matilde Laurá, Menelaos Pipis, Alejandro Horga, James Polke, Diego Kaski, Rita Horvath, Patrick F Chinnery, Wilson Marques, Cristina Tassorelli, Grazia Devigili, Lea Leonardis, Nick W Wood, Adolfo Bronstein, Paola Giunti, Stephan Züchner, Tanya Stojkovic, Nigel Laing, Richard H Roxburgh, Henry Houlden, Mary M Reilly

**Affiliations:** 1 Department of Neuromuscular Disease, UCL Queen Square Institute of Neurology and The National Hospital for Neurology, London, UK; 2 Department of Brain and Behavioral Sciences, University of Pavia, Pavia, Italy; 3 Department of Neuroscience, Reproductive Sciences and Odontostomatology, University of Naples “Federico II”, Naples, Italy; 4 Department of Neurology, Fondazione Policlinico Universitario A. Gemelli IRCSS, Rome, Italy; Institute of Neurology, Catholic University of the Sacred Heart, Rome, Italy; 5 Centre for Medical Research University of Western Australia, Harry Perkins Institute of Medical Research, QEII Medical Centre, Nedlands, Western Australia 6009, Australia; 6 Department of Clinical and Movement Neuroscience, UCL Queen Square Institute of Neurology and The National Hospital for Neurology, London, UK; 7 Auckland District Health Board (ADHB), Auckland, New Zealand; Centre of Brain Research Neurogenetics Research Clinic, University of Auckland, New Zealand; 8 Neurogenetic Unit, Royal Perth Hospital, Perth, West Australia, Australia; 9 Department of Neurology, Wellington Hospital, Wellington 6021, New Zealand; 10 Department of Neurology, Tauranga Hospital, Private Bag, Cameron Road, Tauranga 3171, New Zealand; 11 Sorbonne Université, AP-HP, Hôpital Pitié-Salpêtrière, Department of Neurophysiology, Paris France; 12 CHU Nantes, Reference Centre for Neuromuscular Diseases, Hôtel-Dieu, Nantes, France; 13 Department of Neurology, CHU Bicêtre, AP-HP, Le Kremlin-Bicêtre, France; 14 IRCCS Mondino Foundation, Pavia, Italy; 15 Department of Neurology, School of Medicine of Ribeirão Preto, University of São Paulo, Ribeirão Preto, Brazil; 16 Department of Clinical Neurosciences, University of Cambridge, Cambridge Biomedical Campus, Cambridge, UK; 17 MRC Mitochondrial Biology Unit, University of Cambridge, Cambridge, UK; 18 UO Neurologia I, Fondazione IRCCS Istituto Neurologico “Carlo Besta”, Milano, Italy; 19 Division of Neurology, Institute of Clinical Neurophysiology, University Medical Centre Ljubljana, Ljubljana, Slovenia; 20 Dr. John T. Macdonald Foundation Department of Human Genetics and John P. Hussman Institute for Human Genomics, University of Miami Miller School of Medicine, Miami, Florida, USA; 21 Sorbonne Université, AP-HP, Hôpital Pitié-Salpêtrière, Centre de Référence des Maladies Neuromusculaires, Nord/Est/Ile-de-France, Inserm UMR_S 974, Paris, France; 22 Neurogenetics Unit, Department of Diagnostic Genomics, PathWest Laboratory Medicine, QEII Medical Centre, Nedlands, Australia

**Keywords:** sensory neuropathy, cerebellar ataxia, neuropathy, vestibular areflexia syndrome (CANVAS), chronic cough, RFC1, repeat expansion

## Abstract

Ataxia, causing imbalance, dizziness and falls, is a leading cause of neurological disability. We have recently identified a biallelic intronic AAGGG repeat expansion in replication factor complex subunit 1 (*RFC1*) as the cause of cerebellar ataxia, neuropathy, vestibular areflexia syndrome (CANVAS) and a major cause of late onset ataxia. Here we describe the full spectrum of the disease phenotype in our first 100 genetically confirmed carriers of biallelic repeat expansions in *RFC1* and identify the sensory neuropathy as a common feature in all cases to date. All patients were Caucasian and half were sporadic. Patients typically reported progressive unsteadiness starting in the sixth decade. A dry spasmodic cough was also frequently associated and often preceded by decades the onset of walking difficulty. Sensory symptoms, oscillopsia, dysautonomia and dysarthria were also variably associated. The disease seems to follow a pattern of spatial progression from the early involvement of sensory neurons, to the later appearance of vestibular and cerebellar dysfunction. Half of the patients needed walking aids after 10 years of disease duration and a quarter were wheelchair dependent after 15 years. Overall, two-thirds of cases had full CANVAS. Sensory neuropathy was the only manifestation in 15 patients. Sixteen patients additionally showed cerebellar involvement, and six showed vestibular involvement. The disease is very likely to be underdiagnosed. Repeat expansion in *RFC1* should be considered in all cases of sensory ataxic neuropathy, particularly, but not only, if cerebellar dysfunction, vestibular involvement and cough coexist.

## Teaser

Cortese *et al.* describe the full disease phenotype, including progression of ataxia, in 100 confirmed carriers of *RFC1* repeat expansions. *RFC1* repeat expansion should be considered in all cases of sensory ataxic neuropathy, particularly if cerebellar dysfunction, vestibular involvement and cough coexist.

See Paisán-Ruiz and Jen (doi:10.1093/brain/awaa015) for a scientific commentary on this article.

## Introduction

Imbalance, together with dizziness and falls, is one of the leading causes of neurological disability with an estimated prevalence of up to 30% in adults over 65 years of age (Lin and Bhattacharyya, 2012; Hartholt *et al.*, 2019). A key feature of normal balance function is the cross-modal integration of visual, proprioceptive, and vestibular afferents that also undergo cerebellar modulation. Failure across any of these systems can lead to imbalance with resulting ataxia, but the cause remains elusive in a significant proportion of cases.

We have recently identified a biallelic intronic repeat expansion in replication factor complex subunit 1 (*RFC1*) as a major cause of late onset ataxia (Cortese *et al.*, 2019). The cerebellum, the sensory neurons and the vestibular system can all be affected in this neurodegenerative disorder, which has also been termed cerebellar ataxia, neuropathy, vestibular areflexia syndrome (CANVAS) (Szmulewicz *et al.*, 2011).

Since the original descriptions of this syndrome (Bronstein *et al.*, 1991), there have been a growing number of studies describing the clinical features of CANVAS (Migliaccio *et al.*, 2004; Szmulewicz *et al.*, 2011; Wu *et al.*, 2014; Cazzato *et al.*, 2016; Infante *et al.*, 2018; Pelosi *et al.*, 2018; Taki *et al.*, 2018). Although these descriptions are useful, these reports were limited by the inclusion of patients using purely clinical criteria (Szmulewicz *et al.*, 2016). Therefore, the detailed phenotypic spectrum of *RFC1* repeat expansion has yet to be fully elucidated.

Here we report our first 100 genetically confirmed patients carrying a biallelic AAGGG repeat expansion in *RFC1*. Clinical presentation, phenotypic heterogeneity and progression of the disease are discussed.

## Materials and methods

### Patients

DNA samples (*n = *363) were collected from patients diagnosed with adult-onset ataxia or CANVAS at the National Hospital for Neurology and Neurosurgery at Queen Square (London, UK) and in neurological centres in New Zealand, France, Italy, Brazil, Slovenia Australia and the UK. Clinical data were gathered using a standardized proforma. After reviewing the clinical charts, the following information was collected for all cases tested: demographics, family history, diagnosis, clinical and/or investigational evidence of sensory neuropathy, cerebellar dysfunction/atrophy, vestibular areflexia and the presence/absence of additional atypical features for CANVAS (e.g. associated parkinsonism, spasticity, cognitive decline). Additional information was collected in cases testing positive for biallelic *RFC1* expansions: history of cancer, prior alternative diagnoses, symptoms and age of symptom onset, use of walking aids, first neurological examination available, autonomic testing, previous genetic testing and muscle or nerve biopsy.

Clinical involvement of the main systems affected in *RFC1* repeat expansion associated CANVAS were defined as follows: (i) sensory neuropathy/neuronopathy: sensory symptoms (loss of feeling, paraesthesia, dysesthesia, neuropathic pain) and/or abnormal sensory examination including sensory ataxia; (ii) bilateral vestibulopathy: presence of head movement-induced oscillopsia and/or abnormal head impulse test; and (iii) cerebellar dysfunction: cerebellar dysarthria and/or dysphagia, abnormal eye movements (nystagmus, dysmetric saccades, broken pursuit). Investigational involvement was further defined by (i) sensory neuropathy/neuronopathy on nerve conduction studies; (ii) bilaterally abnormal video head impulse test, bithermal caloric or motorized rotational tests; and (iii) cerebellar atrophy on brain MRI. We defined full CANVAS cases as those with a combined involvement of the sensory peripheral nerves, vestibular system and the cerebellum in the absence of atypical features.

The study was approved by local institutional ethical committees. Written informed consent was obtained from patients, all aged over 18 years, participating in the study.

### *RFC1* pentanucleotide repeat expansion testing

Flanking polymerase chain reaction (PCR) and repeat-primed PCRs for the pathogenic AAGGG and non-pathogenic AAAGG or AAAAG repeat expansions in *RFC1* were performed as previously described (Cortese *et al.*, 2019). Positive cases were defined as samples showing no PCR amplifiable product on flanking PCR, negative repeat primed PCR for (AAAGG)_exp_ and (AAAAG)_exp_ configurations, and the presence of a decremental saw-tooth pattern on repeat primed PCR for the pathogenic AAGGG repeat expansion. If a sufficient amount of DNA was available, Southern blotting was performed according to our previously-described protocol in positive cases to confirm the presence and measure the size of the AAGGG repeat expansions (Cortese *et al.*, 2019).

### MRI

Brain and spine scans were performed according to standard clinical protocols. Axial fluid-attenuated inversion recovery (FLAIR), coronal T_2_-weighted and 3D T_1_ sequences were used to evaluate cerebellar atrophy and sagittal and axial T_2_-weighted images were performed to evaluate spinal cord atrophy.

### Nerve conduction studies

Surface orthodromic motor (median, ulnar, posterior tibial, peroneal nerves) and antidromic (orthodromic for ulnar and median in UK centres) sensory (median, ulnar, radial, sural and/or superficial peroneal nerves) nerve conduction studies were performed according to standard procedures (Preston and Shapiro, 2013). Amplitudes of distal compound muscular action potential and sensory action potential were measured from baseline to negative peak. Potentials in the upper and lower limbs were considered absent when all potentials of nerves tested were not recordable and reduced when at least one potential had reduced amplitude according to the normal value of each centre.

### Vestibular testing

Bilaterally reduced or absent angular vestibulo-ocular reflex function was documented by at least one of the following tests (Strupp *et al.*, 2017).

#### Bilaterally abnormal video head impulse test

The horizontal angular vestibulo-ocular reflex velocity gain (the ratio of eye velocity to head velocity) was quantified by a video-oculography system and an accelerometer that measured eye and head velocity during passive, unpredictable, impulsive (10°–10° amplitude, 150–300°/s acceleration) head turns delivered by the examiner in the plane of the horizontal canal in both directions (left and right), while subjects were required to stare at a head fixed target at eye level (Bronstein *et al.*, 2015).

#### Bilaterally reduced caloric response

The low frequency angular vestibulo-ocular reflex was tested using a video-oculography system and an irrigation unit. The horizontal canal of each ear was irrigated with cold (30°C) and warm (44°C) water. Eye movements were recorded after each irrigation and the maximal slow-phase velocity of caloric induced nystagmus was measured (Cousins *et al.*, 2013).

#### Reduced vestibulo-ocular reflex gain tested using a rotatory chair

Peak slow-phase eye velocity was recorded by a video-oculography system while the subjects were rotated on a motorized chair. The chair was rapidly accelerated (1 s) in the yaw plane to a constant velocity of 90°/s using a rotational step stimulus (Okada *et al.*, 1999). Vestibulo-ocular reflex gain (the ratio of eye velocity to chair velocity during sinusoidal chair rotation in the range from 0.05 to 0.1 Hz) and time constant (the rate of decay of slow phase eye velocity during constant velocity steps rotation) were recorded.

### Autonomic assessment

Parasympathetic function was assessed by ECG monitoring of heart rate variation during Valsalva manoeuvre, deep breathing and standing (Ewing *et al.*, 1985). Sympathetic function was assessed by measuring blood pressure response to change in posture and handgrip and sympathetic skin response. Parasympathetic or sympathetic function were considered impaired if at least one test was definitely abnormal.

### Neuropathological examination

Nerve biopsies were processed according to local standard operating procedures. In brief, fresh biopsies, immediately after removal, were divided in half, with one portion fixed in 10% buffered formalin, followed by tissue processing and embedding in paraffin and the other portion fixed in 3% glutaraldehyde, followed by processing into epoxy resin. The paraffin embedded tissues were cut at 5-µm thin sections and stained with a battery of histochemical and immunohistochemical stains (including immunostaining for neurofilaments with SMI31 antibody (1:5000; Sternberger, shown in Fig. 3) and the resin-embedded tissues were cut into 1-µm semi-thin sections and stained with methylene blue azure-basic fuchsin. All slides were viewed under light microscope. Histology images shown in the Fig. 3 were digitized on a LEICA SCN400F scanner at ×40 magnification and 65% image compression setting during export. For the preparation of light microscopy images, image captures were taken in LEICA SlidePath (LEICA Milton Keynes, UK). Publication figures were assembled in Adobe® Photoshop.

For the analysis of myelinated fibre density in sural nerve biopsies, four randomly selected fascicles from each case were assessed. Both large and small myelinated fibres were counted.

### Statistical analyses

Continuous data are expressed as median (minimum–maximum). Spearman’s correlation coefficient was calculated to test the association of repeat expansion size and age of neurological onset, age at onset of unsteadiness, time-to-use of a stick and disease severity, as indicated by the number of systems involved (cerebellar, peripheral nerve, vestibular system). Mann-Whitney test was used to compare the repeat expansion size in patients with and without history of cancer. All analyses were performed using STATA statistical software, version 14.

### Data availability

Anonymized data from this study will be shared by request from any qualified investigator.

## Results

### Genetic screening for *RFC1* AAGGG expansion

From the 363 recruited cases, we identified 105 patients carrying the biallelic AAGGG repeat expansion in *RFC1*. We have reported limited clinical details of 56 cases in our original paper describing the *RFC1* repeat expansion (Cortese *et al.*, 2019) and add here 49 further cases identified by screening additional DNA samples.

Overall, the biallelic AAGGG repeat expansion in *RFC1* was identified in 63/70 (90%) of DNA samples from patients with full CANVAS and 42/293 (14%) of DNA samples from a range of other adult onset ataxia cases.

In 51 cases for which sufficient DNA was available, Southern blotting was performed confirming the presence of repeat expansions of both alleles in all cases. Expansion size ranged from 433 to 2750 repeat units, with a median size of 989 repeats for the smaller allele and 1802 for the larger allele. In 24 cases the expanded alleles had similar size as shown by the presence of one discrete band on Southern blot.

### Patient demographics and previous diagnoses

Detailed clinical information was available from 100 of 105 genetically confirmed cases, which are considered here for further analysis.

There were 45 male and 55 female patients. Forty-five cases from 26 families had a positive family history with at least one sibling affected or an affected cousin (three families), while in 55 cases the disease was sporadic. Median age at the time of this study was 72 years (range 45–95). All patients were Caucasian.

Fifty-one cases were routinely seen in peripheral nerve/neuromuscular clinics, 16 in general neurology clinics, 20 in neurogenetic clinics, seven in ataxia clinics and six in neuro-otology clinics.

CANVAS was the initial diagnosis in only 23 cases. Twenty-two cases were considered to have a pure sensory neuropathy or neuronopathy; 23 further patients were defined as having a complex neurological disorder but not actual CANVAS, including complex neuropathy (*n = *14), complex ataxia (*n = *7) or complex vestibular syndromes (*n = *2), depending on the predominant system involved. Thirty-two patients received an alternative likely diagnosis in the first instance including spino-cerebellar ataxia (*n = *16), mitochondrial disease (*n = *6), multisystem atrophy-cerebellar subtype (*n = *3), cerebrovascular disease (*n = *2), hereditary sensory neuropathy (*n = *2), hereditary spastic paraplegia (*n = *1), paraneoplastic disease (*n = *1) and Sjögren’s syndrome (*n = *1).

### Past medical history

As the repeat-hosting gene *RFC1* encodes for the largest subunit of the replication factor complex, essential to DNA replication and repair, detailed data concerning cancer history were also collected. Twelve patients reported a positive history of cancer as follows: breast (*n = *5), prostate (*n = *1), gastric (*n = *1), lymphoma (*n = *1), parathyroid (*n = *1), metastatic disease with unknown primary (*n = *1), basal cell carcinoma (*n *= 1), melanoma and bowel adenocarcinoma in the same patient. This was similar to the 14% of positive neoplasm history observed in 100 randomly chosen ataxia patients from the National Hospital for Neurology and Neurosurgery, Queen Square (London, UK), with the same age and gender distribution as *RFC1* positive cases and who tested negative for *RFC1* repeat expansion (*P = *0.69).

### Symptoms at onset and during disease progression

**Figure 1:**
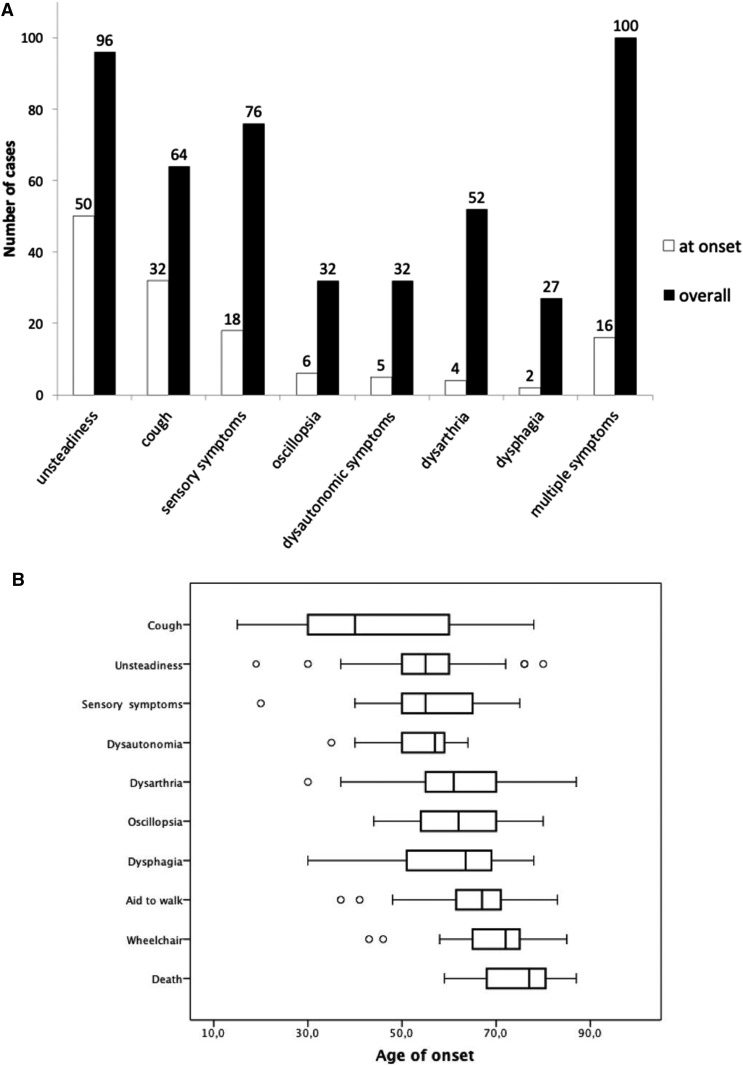
**Disease onset and progression.** (**A**) Symptoms at disease onset and during disease progression in 100 biallelic RFC1 expansion carriers. Patients reporting a combination of symptoms (unsteadiness, cough, sensory symptoms, oscillopsia, dysautonomia, dysarthria, dysphagia) are also represented in the last two columns (multiple symptoms). (**B**) Age distribution of symptoms and disease milestones. The ends of the boxes represent the upper and lower quartiles, the median is marked by a vertical line within the box. The whiskers extend to the highest and lowest observations with outliers represented as circles outside the whiskers.

Median age of any neurological onset of the disease (cough excluded) was 52 years, ranging from 19 to 76 years. Frequency of symptoms and age distribution of their onset are summarized in Fig. 1A and B. Detailed clinical information for each case is provided in Supplementary Table 1.

Progressive imbalance was reported by 96 patients, at a median age of 55 years (range 30–80), and was considered the presenting symptom in 50 patients.

Seventy-six patients complained of sensory symptoms, including loss of feeling (*n = *52), pins and needles (*n = *27), neuropathic pain (*n = *42). In 18 cases sensory symptoms were reported at disease onset.

Oscillopsia, defined as a visual disturbance in which objects appear to oscillate during head movements, is a common sequela of a bilaterally impaired vestibulo-ocular reflex, and was reported by 32 patients and in six at disease onset.

Dysarthria and dysphagia, secondary to cerebellar dysfunction, were reported in 52 and 27 patients, respectively, but were rarely the presenting symptom. Rather, they tended to appear later in the disease course.

Symptoms of autonomic dysfunction, including postural hypotension (*n = *13), erectile dysfunction (*n = *7), chronic constipation (*n = *7), urinary dysfunction (*n = *8) and anhidrosis (*n = *3) were reported by 32 patients, but these were usually mild and tended to appear late.

Notably, 64 patients complained of chronic cough, which could precede the onset of neurological symptoms by three decades and was the initial symptom in a third of all cases.

The disease had a slowly progressive course. Fifty-five patients needed a stick after 10 years of disease duration (range 0–42) and 25 were wheelchair-dependent after 15 years of disease duration (range 2–38). At the time of this study 12 patients had died. Median age of death was 77 years (range 59–87).

We did not observe a significant correlation between the size of the smaller allele, larger allele or their sum and age of neurological onset (cough excluded), age at onset of unsteadiness, time-to-use of a stick or disease severity as indicated by number of systems involved (Supplementary Fig. 1). Also, there was no difference in repeat expansion size between patients with and without history of cancer.

### Neurological examination

Neurological examination was performed after 10 (0–50) years from recorded neurological disease onset (Table 1). Seventy-nine cases had ataxic gait and 83 had a positive Romberg’s sign. Eye movement examination was abnormal in 65 cases, showing nystagmus (*n = *55), broken pursuit (*n = *42) or dysmetric saccades (*n = *41), suggesting the presence of an underlying cerebellar dysfunction. Dysarthric speech, although understandable, was noted in 40 patients. Bedside head impulse test was performed in 32 cases and showed bilateral vestibular hypofunction (catch-up saccades) in 28 of them (87%) (Supplementary Video 1). Pinprick sensation was reduced in the face in 22, in the upper limbs in 55, in the lower limbs in 74 patients. Vibratory sensation was abnormal in the upper limbs in 41 and in the lower limbs in 84 cases. Joint position was generally preserved in the upper limbs but abnormal in less than half of the patients in the lower limbs. Muscle bulk and strength were preserved, while tone was reduced in seven cases. Deep tendon reflexes in the upper limbs and patellar reflex in the lower limbs were normally elicitable in half, reduced or absent in a quarter and brisk in another quarter of cases. Ankle reflexes were more frequently reduced or absent but retained or even brisk ankle jerks were observed in 45 cases. Plantar responses were flexor in all patients.

**Table 1:** Patients demographics and neurological examination

	*n* = 100
**Demographics**	
Male	45
Positive family history	45
Age of onset	52 (range 19–76)
Age at examination	63 (range 33–82)
History of cancer	12
Previous misdiagnosis	32
**Neurological examination**	79
Ataxic gait	
Positive Romberg	83
Dysarthria	40
Abnormal eye movements	65
Nystagmus	55
Broken pursuit	42
Dysmetric saccades	41
Abnormal head impulse test	28/32 (87%)
**Sensation**	
Abnormal pinprick face	22
Abnormal pinprick upper limbs^a^	55
Abnormal pinprick lower limbs^b^	74
Abnormal vibration upper limbs	41
Abnormal vibration lower limbs	84
Abnormal joint position upper limbs	10
Abnormal joint position lower limbs	48
**Muscle strength**	
Distal weakness upper limbs	0
Distal weakness lower limbs	0
**Reflexes**	
Upper limb reflexes	
Normal	54
Brisk	26
Reduced	12
Absent	8
Knee reflexes	
Normal	47
Brisk	28
Reduced	13
Absent	12
Ankle reflexes	
Normal	30
Brisk	15
Reduced	6
Absent	49
**Coordination**	
Abnormal finger-nose	49
Abnormal heel-shin	52

a Absent = 12/55 (22%), patchy = 2/55 (4%), length dependent pattern *n *= 41/55 (74%).

b Absent = 13/74 (18%), patchy = 4/74 (5%), length dependent pattern *n *= 57/74 (77%).

### Investigations

Results of investigations performed are summarized in Table 2. Nerve conduction study data were available in 95 patients. Sensory conduction was invariably abnormal, with sensory action potentials either reduced or absent in the upper limbs and usually absent in the lower limbs. Conduction velocities were either normal or reduced within the range expected based on sensory fibre loss. Motor conduction was normal or mildly reduced consistent with axonal loss. Compound muscle action potentials were considered reduced for age in the lower limbs in 10 cases and in the upper limbs in two cases.

**Table 2:** Investigations

Investigation	*n*/available data (%)
Abnormal sensory nerve conduction study	95/95 (100)
Sensory action potential upper limbs	
Reduced	40/92 (45)
Absent	52/92 (57)
Sensory action potential lower limbs	
Reduced	15/90 (17)
Absent	75/90 (83)
Reduced amplitude of compound motor action potential in upper limbs	2/94 (2)
Reduced amplitude of compound motor action potential in lower limbs	10/93 (11)
Spinal cord atrophy on cervical MRI	19/42 (45)
Posterior column changes on cervical MRI	4/34 (12)
Cerebellar atrophy on brain MRI^a^	57/91 (63)
Cerebral atrophy on brain MRI^a^	17/91(19)
White matter changes on brain MRI	31/91 (34)
Bilateral vestibular impairment	48/53 (91)
Autonomic dysfunction	
Parasympathetic dysfunction^b^	9/36 (25)
Sympathetic dysfunction^c^	11/32 (34.4)
Combined parasympathetic and sympathetic dysfunction	10/42 (23.8)

a One patient only had brain CT scan.

b Heart rate during Valsalva manoeuvre, deep breath and standing.

c Blood pressure during change in posture and handgrip; sympathetic sudomotor response.

Spine MRI was performed in 42 cases and showed cord atrophy and T_2_ hyperintensity in the posterior column in 45% (*n = *19) and 12% (*n = *4), respectively.

Cerebellar atrophy, mainly involving the vermis, was observed in 63% (*n = *57) of 91 cases who underwent brain MRI. A third of cases also had supratentorial white matter changes, of putative microvascular origin.

Vestibular testing was performed in 53 cases and showed the presence of bilateral vestibular hypofunction in 91% of them (*n = *48), including three cases with normal bedside head impulse test.

Autonomic testing was performed in 42 cases and was abnormal in half, showing either a primary sympathetic (*n = *11/32, 34%), parasympathetic (*n = *9/36, 25%) or mixed sympathetic and parasympathetic (*n = *10/42, 24%) dysfunction.

Patients underwent multiple additional investigations to rule out other acquired or hereditary disorders. In particular, a muscle biopsy was performed in 16 cases in which a mitochondrial disease was suspected and was either normal or showed mild non-specific changes. Fifteen cases underwent a nerve biopsy to rule out an inflammatory or vasculitic cause of the neuropathy. Nerve biopsies typically showed a severe chronic loss of large and small myelinated fibres without any active axonal degeneration and no regeneration (Fig. 3). In eight cases sural nerve biopsy was reviewed to quantify fibre loss. Myelinated fibre nerve density was severely reduced (median 290 myelinated fibres per mm^2^ cross-sectional area, ranging from 52 to 2067) compared to age-matched controls [normal range 4000–9000 myelinated fibres/mm^2^ (Ochoa and Mair, 1969; Jacobs and Love, 1985)]. There was no significant variation of fibre density across the fascicles (median 28 myelinated fibres per fascicle, ranging from 4 to 81). The fibre loss was not accompanied by any significant regeneration and there was no active axonal degeneration. There was also no evidence of demyelination.

Extensive genetic testing was performed and was negative including Friedreich’s ataxia repeat expansion (*n = *56), common SCAs repeat expansions (*n = *54), mitochondrial DNA sequencing (*n = *37), DNA polymerase gamma (*POLG*) sequencing (*n = *5) and next-generation sequencing targeted panels encompassing genes associated with neuropathy and/or ataxia (*n = *25).

### Multisystem involvement of *RFC1* repeat expansion

**Figure 2:**
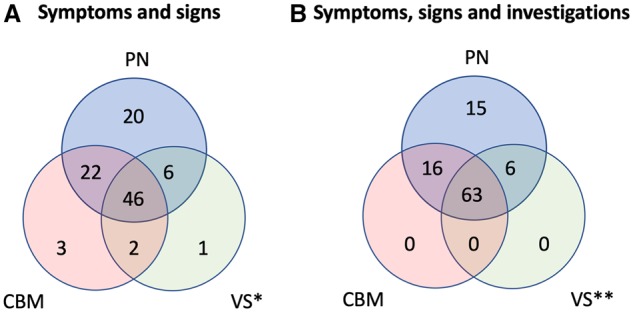
**Involvement of peripheral nerve, cerebellum and vestibular system in *RFC1* biallelic expansion.** (**A**) Symptoms and signs. Clinical involvement of peripheral nerve (PN), vestibular system (VS) and cerebellum (CBM) is defined as follows: (i) peripheral nerve: sensory neuropathy: sensory symptoms (loss of feeling, paraesthesia, dysesthesia, neuropathic pain) and/or abnormal sensory examination including sensory ataxia; (ii) vestibular system: bilateral vestibulopathy: presence of head movement-induced oscillopsia and/or abnormal head impulse test; (iii) cerebellum: cerebellar dysfunction: cerebellar dysarthria and/or dysphagia, abnormal eye movements (nystagmus, dysmetric saccades, broken pursuits). (**B**) Symptoms, signs and investigations. In addition to the clinical involvement as defined in **A**, investigational involvement of the three main system affected in CANVAS was further identified by (i) peripheral nerve: sensory neuropathy/neuronopathy on nerve conduction studies; (ii) vestibular system: bilaterally abnormal video head impulse test, bithermal caloric or motorized rotational tests; and (iii) cerebellum: cerebellar atrophy on brain MRI. *Abnormal in 55 of 59 (93%) with clinical information available. **Abnormal in 69 of 74 (93%) with clinical and/or investigational information available.

Based on symptoms and neurological examination, 94 patients had clinical evidence of sensory neuropathy/neuronopathy, 73 of cerebellar dysfunction and 55 of vestibular impairment (Fig. 2A). After investigations, two-thirds of the patients showed involvement of the three main systems implicated in CANVAS. A subclinical sensory neuropathy was identified in six cases confirming a sensory neuropathy in 100% of cases, cerebellar atrophy in six patients without clinical evidence of cerebellar dysfunction and subclinical vestibular impairment in an additional 14 patients. In 15 cases an isolated sensory neuropathy remained the only manifestation of the disease (Fig. 2B). However, video head impulse test was performed (normal) in only two of them. Therefore, subclinical vestibular involvement cannot be excluded in patients with isolated sensory neuropathy.

**Figure 3:**
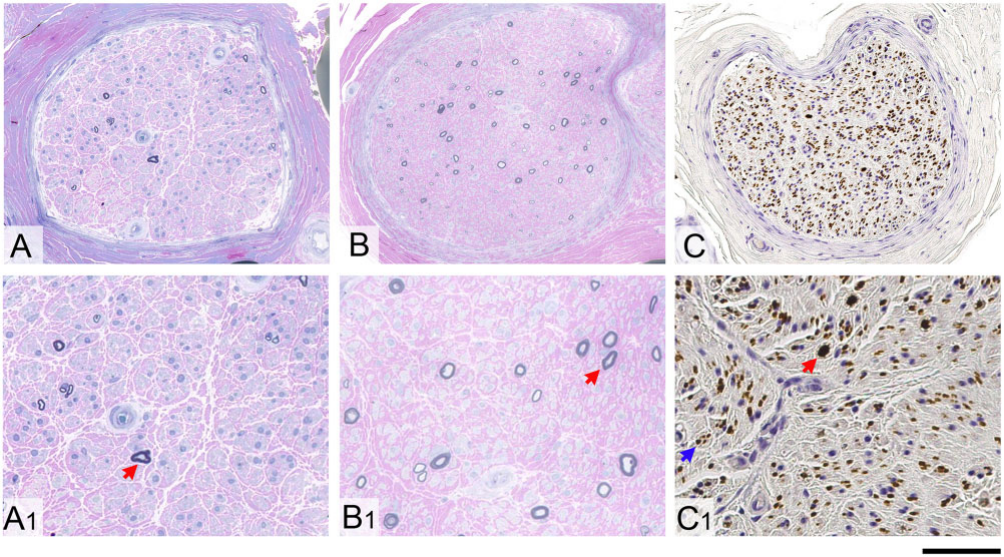
**Nerve biopsy findings in RFC1 biallelic repeat expansions.** Severe loss of large and small myelinated fibres with no evidence of ongoing axonal degeneration, no signs of regeneration and no features of demyelination is seen in all CANVAS patients with sensory neuropathy due to *RFC1* biallelic expansion. (**A**, **A1**, **B** and **B1**) Resin-embedded, semi-thin sections stained with methylene blue azure-basic fuchsin are shown from two different patients. Occasional large remaining fibres in both cases are highlighted with a red arrow in **A1** and **B1**. The unmyelinated fibres in the peripheral nerves from CANVAS patients are comparably much better preserved. (**C**) Axons in formalin-fixed paraffin-embedded tissue are shown on immunostaining with SMI31 antibody. (**C1**) A large residual axon is highlighted with red arrow and one of multiple clusters of small unmyelinated fibres is shown with a blue arrow. Scale bars = 100 µm in **A**–**C**; 40 µm in **A1**–**C1**.

## Discussion

This paper explores the phenotypic spectrum of biallelic AAGGG repeat expansions in *RFC1* in our first 100 genetically confirmed patients.

The cases were enrolled from multiple centres across three continents, however, despite the ethnically heterogeneous populations living in these countries, all genetically confirmed patients are Caucasian, and only ∼50% reported a positive family history for the disease.

Two-thirds of genetically confirmed cases had full CANVAS after clinical examination and investigations; a complex sensory ataxia with cerebellar or vestibular involvement was identified in 16 and six, respectively, while in 15 a sensory neuropathy was the only clinically detectable manifestation of the disease. None had isolated cerebellar syndrome or pure bilateral vestibular failure. The disease is slowly progressive, but often disabling with half of the patients needing a stick after 10 years of disease duration and a fourth being wheelchair dependent after 15 years.

The disease seems to follow a pattern of spatial progression from the early involvement of sensory neurons, to the later appearance of vestibular and cerebellar dysfunction. This is supported by: (i) the progression of symptoms, from gait ataxia (median onset 55 years, range 30–80) and distal sensory complaints (median onset 55 years, range 20–75), to the later appearance of oscillopsia and visual disturbances (median onset 62 years, range 44–80), dysarthria (median onset 61, range 30–87) and dysphagia (median onset 64 years, range 30–78); (ii) the higher frequency of peripheral neuropathy as detected by nerve conduction studies (∼100%) compared to vestibular failure (∼80%) and cerebellar dysfunction (∼60%) in the first available cross-sectional examination; (iii) the higher frequency of peripheral neuropathy (100%) compared to vestibular failure (93%, 69 of 74 tested) and cerebellar dysfunction (79%) (Fig. 2B); and (iv) the presence (∼20%) of pure neuropathic/neuronopathic forms as opposed to the absence of isolated cerebellar or vestibular variants.

As sensory neuropathy is constant and pivotal to the neurodegenerative process, additional considerations are made here to characterize it further. In contrast to some other hereditary sensory neuropathies, patients with biallelic *RFC1* expansions do not exhibit insensitivity to pain, with painless injuries, ulceration and amputations. This suggests that Aδ and C fibre sensory neurons are selectively spared. We postulate that large-diameter sensory neurons innervating muscle spindles are also relatively spared, explaining the frequent occurrence of normal, if not brisk, deep tendon reflexes in the context of a progressive loss of Aα and Aβ myelinated fibre sensory neurons, controlling superficial sensation and proprioception. This observation is consistent with previous reports of preserved H reflexes in the upper and lower limbs of CANVAS patients (Burke and Halmagyi, 2018; Infante *et al.*, 2018).

Moreover, the observation of atrophy and T_2_-weighted high signal intensity in the posterior columns on spine MRI, together with the current findings on nerve conduction studies and previous ultrasound detection of small diameter nerves (Pelosi *et al.*, 2018), concordantly indicate that a sensory neuronopathy, with combined degeneration of central and peripheral projections of the T-shaped dorsal root ganglion neurons, is part of the neurodegenerative process in *RFC1* biallelic expansions (Asbury, 1987; Lauria *et al.*, 2000; Camdessanché *et al.*, 2009). The primary involvement of sensory neurons is further corroborated by the absence of significant regeneration on nerve biopsies in the present study and confirms previous pathological observations from two post-mortem CANVAS cases of subtotal neuronal loss in the dorsal root ganglia, without satellite cell proliferation (Szmulewicz *et al.*, 2014)

Vestibular symptoms, including oscillopsia or more non-specific visual disturbances while walking or during head movements, were reported in a third of cases. Oscillopsia in some cases was so severe that fixation and face recognition became impossible during self-motion, typically walking or travelling in cars. In contrast, rotational vertigo was not a common complaint. However, this is not surprising as rotational vertigo stems from an asymmetric dysfunction of vestibular systems, and its absence in CANVAS syndrome further supports the presence of a bilateral symmetrical degenerative process. Subclinical vestibular involvement was detected in an additional ∼30% cases if specifically looked for, and it is likely to be underestimated in the present series since bedside or video head impulse test were performed only in half of the cases.

Two-thirds of patients had clinical and MRI evidence of cerebellar involvement. In the context of severe sensory ataxia, abnormal eye movements were often the first clue to cerebellar involvement and were accompanied by dysarthria and dysphagia in the later stages of the disease course.

An unexplained spasmodic dry cough was present in over 60% of patients and should be considered as part of this complex neurodegenerative disease. Coughing, usually occurring without a specific trigger, was reported up to three decades before onset of difficulty walking and unsteadiness, suggesting a particular susceptibility of neuronal circuits controlling the cough reflex.

The disease-causing mechanisms of intronic *RFC1* expansions are still unknown. In our previous work we did not detect reduced expression of *RFC1* at either transcript or protein level (Cortese *et al.*, 2019). However a more subtle, tissue- or time-specific loss-of-RFC1 function could not be excluded (Cortese *et al.*, 2019). A higher incidence of neoplasm might have been expected as the result of *RFC1* dysfunction together with impaired DNA damage response. Our results reported here and our previous work (Cortese *et al.*, 2019) do not support this hypothesis. This, together with completely normal development, suggests that known *RFC1* biological functions are grossly preserved.

As the allele frequency of *RFC1* AAGGG expansion ranges from ∼1 to 5% of the healthy population (Cortese *et al.*, 2019; Rafehi *et al.*, 2019), the estimated prevalence at birth of biallelic carriers ranges from 1:10 000 to 1:400 individuals, suggesting that the disease is likely to be largely underdiagnosed and, possibly, misdiagnosed. Indeed, CANVAS was initially suspected in only 23% of the patients in the current series and one-third of cases were considered most likely to have an alternative diagnosis including spino-cerebellar ataxia, mitochondrial disease, multisystem atrophy-cerebellar subtype, or an acquired autoimmune condition. This may partly be explained by the relatively recent description of the clinical syndrome (Szmulewicz *et al.*, 2011). Patients frequently had costly and/or invasive investigations including multiple genetic tests and nerve and muscle biopsies. The availability of a diagnostic test for CANVAS will ensure that patients receive a timely diagnosis.

Testing for the *RFC1* repeat expansion should be considered in all cases with the full CANVAS phenotype as well as in cases with sensory neuropathy, particularly, but not only, if cerebellar, vestibular involvement and/or unexplained chronic cough coexist. The study highlights the importance of a thorough clinical examination including bedside head impulse testing in any patients with ataxia regardless of whether the ataxia is considered to be due to a neuropathy or cerebellar dysfunction.

## Supplementary Material

awz418_Supplementary_Materials

## Abbreviation

CANVAS =: cerebellar ataxia, neuropathy, vestibular areflexia syndrome

## References

[awz418-B1] Asbury AK Sensory neuronopathy. Semin Neurol 1987; 7: 58–66. 3332448 10.1055/s-2008-1041406

[awz418-B2] Bronstein AM, Mossman S, Luxon LM The neck-eye reflex in patients with reduced vestibular and optokinetic function. Brain 1991; 114: 1–11. 1998877

[awz418-B3] Bronstein AM, Patel M, Arshad Q A brief review of the clinical anatomy of the vestibular-ocular connections-how much do we know? Eye 2015; 29: 163–70. 25412719 10.1038/eye.2014.262 PMC4330278

[awz418-B4] Burke D, Halmagyi GM Normal tendon reflexes despite absent sensory nerve action potentials in CANVAS: a neurophysiological study. J Neurol Sci 2018; 387: 75–9. 29571876 10.1016/j.jns.2018.01.023

[awz418-B5] Camdessanché J-P, Jousserand G, Ferraud K, Vial C, Petiot P, Honnorat J, et al The pattern and diagnostic criteria of sensory neuronopathy: a case-control study. Brain 2009; 132: 1723–33. 19506068 10.1093/brain/awp136 PMC2702838

[awz418-B6] Cazzato D, Bella ED, Dacci P, Mariotti C, Lauria G Cerebellar ataxia, neuropathy, and vestibular areflexia syndrome: a slowly progressive disorder with stereotypical presentation. J Neurol 2016; 263: 245–9. 26566912 10.1007/s00415-015-7951-9

[awz418-B7] Cortese A, Simone R, Sullivan R, Vandrovcova J, Tariq H, Yau WY, et al Biallelic expansion of an intronic repeat in RFC1 is a common cause of late-onset ataxia. Nat Genet 2019; 51: 649–58. 30926972 10.1038/s41588-019-0372-4 PMC6709527

[awz418-B8] Cousins S, Kaski D, Cutfield N, Seemungal B, Golding JF, Gresty M, et al Vestibular perception following acute unilateral vestibular lesions. PloS One 2013; 8: e61862. 23671577 10.1371/journal.pone.0061862 PMC3650015

[awz418-B9] Ewing DJ, Martyn CN, Young RJ, Clarke BF The value of cardiovascular autonomic function tests: 10 years experience in diabetes. Diabetes Care 1985; 8: 491–8. 4053936 10.2337/diacare.8.5.491

[awz418-B10] Hartholt KA, Lee R, Burns ER, van Beeck EF Mortality from falls among US adults aged 75 years or older, 2000-2016. JAMA 2019; 321: 2131–3. 31162561 10.1001/jama.2019.4185 PMC6549288

[awz418-B11] Infante J, García A, Serrano-Cárdenas KM, González-Aguado R, Gazulla J, de Lucas EM, et al Cerebellar ataxia, neuropathy, vestibular areflexia syndrome (CANVAS) with chronic cough and preserved muscle stretch reflexes: evidence for selective sparing of afferent Ia fibres. J Neurol 2018; 265: 1454–62. 29696497 10.1007/s00415-018-8872-1

[awz418-B12] Jacobs JM, Love S Qualitative and quantitative morphology of human sural nerve at different ages. Brain 1985; 108: 897–924. 4075078 10.1093/brain/108.4.897

[awz418-B13] Lauria G, Pareyson D, Grisoli M, Sghirlanzoni A Clinical and magnetic resonance imaging findings in chronic sensory ganglionopathies. Ann Neurol 2000; 47: 104–9. 10632108

[awz418-B14] Lin HW, Bhattacharyya N Balance disorders in the elderly: epidemiology and functional impact. Laryngoscope 2012; 122: 1858–61. 22645067 10.1002/lary.23376

[awz418-B15] Migliaccio AA, Halmagyi GM, McGarvie LA, Cremer PD Cerebellar ataxia with bilateral vestibulopathy: description of a syndrome and its characteristic clinical sign. Brain 2004; 127: 280–93. 14607788 10.1093/brain/awh030

[awz418-B16] Ochoa J, Mair WG The normal sural nerve in man. I. Ultrastructure and numbers of fibres and cells. Acta Neuropathol 1969; 13: 197–216. 5805973 10.1007/BF00690642

[awz418-B17] Okada T, Grunfeld E, Shallo-Hoffmann J, Bronstein AM Vestibular perception of angular velocity in normal subjects and in patients with congenital nystagmus. Brain 1999; 122: 1293–303. 10388795 10.1093/brain/122.7.1293

[awz418-B18] Pelosi L, Mulroy E, Leadbetter R, Kilfoyle D, Chancellor AM, Mossman S, et al Peripheral nerves are pathologically small in cerebellar ataxia neuropathy vestibular areflexia syndrome: a controlled ultrasound study. Eur J Neurol 2018; 25: 659–65. 29316033 10.1111/ene.13563

[awz418-B19] Preston DC, Shapiro BE, Electromyography and neuromuscular disorders: clinical-electrophysiologic correlations, 3rd ed: London; New York: Elsevier Saunders; 2013.

[awz418-B20] Rafehi H, Szmulewicz DJ, Bennett MF, Sobreira NLM, Pope K, Smith KR, et al Bioinformatics-based identification of expanded repeats: a non-reference intronic pentamer expansion in RFC1 causes CANVAS. Am J Hum Genet 2019; 105: 151–65. 31230722 10.1016/j.ajhg.2019.05.016 PMC6612533

[awz418-B21] Strupp M, Kim J-S, Murofushi T, Straumann D, Jen JC, Rosengren SM, et al Bilateral vestibulopathy: diagnostic criteria Consensus document of the Classification Committee of the Bárány Society. J Vestib Res 2017; 27: 177–89. 29081426 10.3233/VES-170619 PMC9249284

[awz418-B22] Szmulewicz DJ, McLean CA, Rodriguez ML, Chancellor AM, Mossman S, Lamont D, et al Dorsal root ganglionopathy is responsible for the sensory impairment in CANVAS. Neurology 2014; 82: 1410–5. 24682971 10.1212/WNL.0000000000000352 PMC4001192

[awz418-B23] Szmulewicz DJ, Roberts L, McLean CA, MacDougall HG, Halmagyi GM, Storey E Proposed diagnostic criteria for cerebellar ataxia with neuropathy and vestibular areflexia syndrome (CANVAS). Neurol Clin Pract 2016; 6: 61–8. 26918204 10.1212/CPJ.0000000000000215 PMC4753833

[awz418-B24] Szmulewicz DJ, Waterston JA, MacDougall HG, Mossman S, Chancellor AM, McLean CA, et al Cerebellar ataxia, neuropathy, vestibular areflexia syndrome (CANVAS): a review of the clinical features and video-oculographic diagnosis. Ann N Y Acad Sci 2011; 1233: 139–47. 21950986 10.1111/j.1749-6632.2011.06158.x

[awz418-B25] Taki M, Nakamura T, Matsuura H, Hasegawa T, Sakaguchi H, Morita K, et al Cerebellar ataxia with neuropathy and vestibular areflexia syndrome (CANVAS). Auris Nasus Larynx 2018; 45: 866–70. 29089158 10.1016/j.anl.2017.10.008

[awz418-B26] Wu TY, Taylor JM, Kilfoyle DH, Smith AD, McGuinness BJ, Simpson MP, et al Autonomic dysfunction is a major feature of cerebellar ataxia, neuropathy, vestibular areflexia ‘CANVAS’ syndrome. Brain 2014; 137: 2649–56. 25070514 10.1093/brain/awu196

