# Supplementary material for: Cerebellar ataxia, neuropathy, vestibular areflexia syndrome due to RFC1 repeat expansion
Source: Brain. 2020 Feb 10;143(2):480–90. doi: 10.1093/brain/awz418 (PMC7009469; doi:10.1093/brain/awz418)

Supplementary Figure 1. Repeat expansion size, disease onset and progression. The time to the use of a stick is calculated from the onset of unsteadiness.

Supplementary Figure 1. Repeat expansion size, disease onset and progression

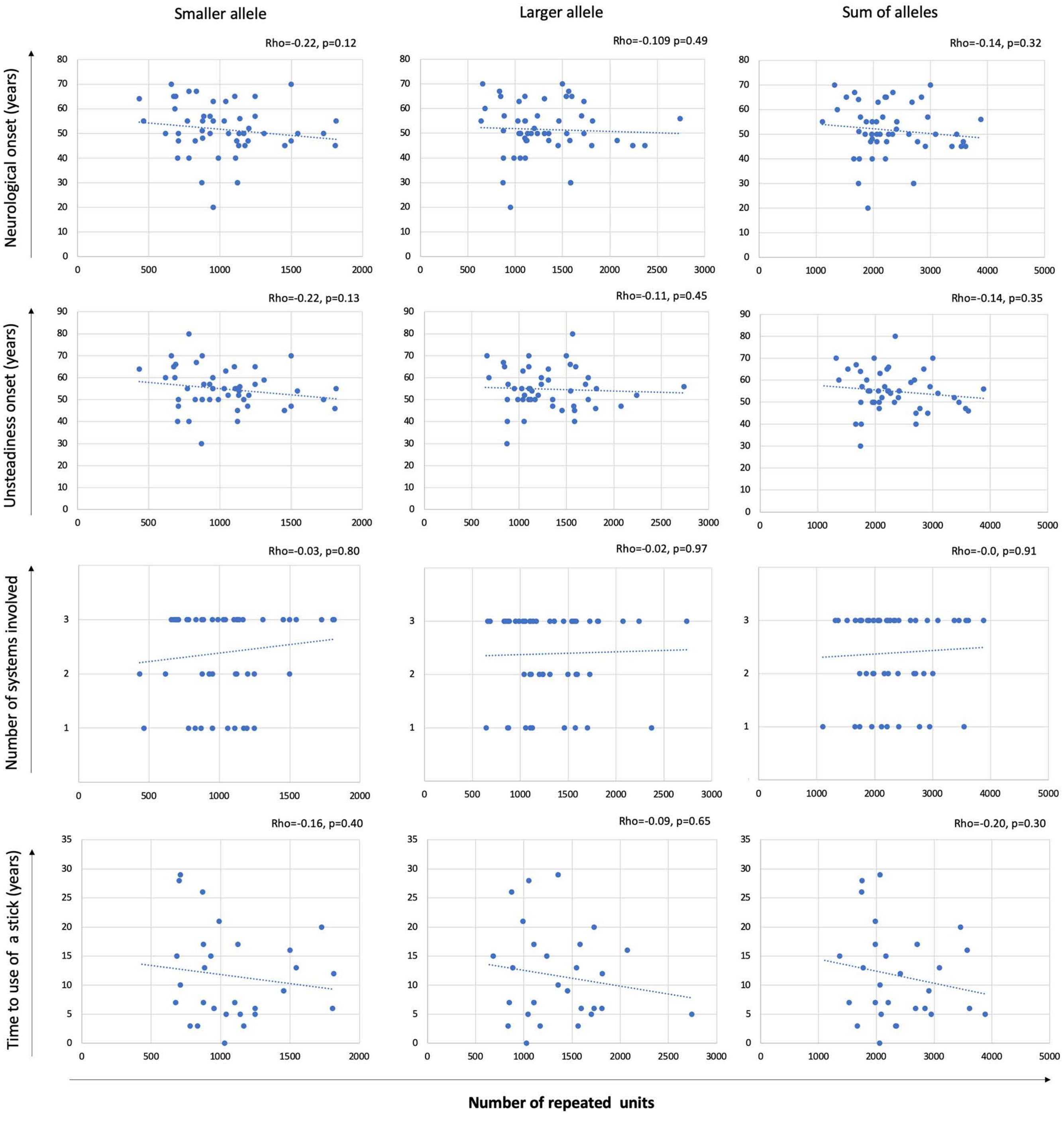

Supplement: awz418_Supplementary_Materials [file awz418_supplementary_materials.zip › awz418-suppl_data/Supplementary Figure 1.pdf]
