# Supplementary material for: Cerebellar ataxia, neuropathy, vestibular areflexia syndrome due to RFC1 repeat expansion
Source: Brain. 2020 Feb 10;143(2):480–90. doi: 10.1093/brain/awz418 (PMC7009469; doi:10.1093/brain/awz418)
Supplement: awz418_Supplementary_Materials [file awz418_supplementary_materials.zip › awz418-suppl_data/Supplementary Table 1.pdf]

Supplementary Table 1 Clinical features of patients with biallelic *RFC1* expansion

ID	Nationality	Current age	Age at examination	Family history	Main clinic for follow-up	Symptom of neurological onset (age)	Sensory peripheral neuropathy	Cerebellar dysfunction	Bilateral vestibular areflexia	Dysautonomia	Cough
1	British	74	63	Family #01	Neuromuscular	Unsteadiness (40)	Yes	Yes	Yes	No	Yes
2	British	76	65	Family #01	Neuromuscular	Unsteadiness (50)	Yes	Yes	Yes	NA	No
3	British	80	69	Family #01	Neuromuscular	Unsteadiness (47)	Yes	Yes	Yes	NA	No
4	British	Died (61)	51	Family #02	Neurogenetics	Dysphagia (30)	Yes	Yes	NA	NA	No
5	British	69	55	Family #02	Neurogenetics	Dysphagia (30)	Yes	Yes	Yes	No	No
6	British	Died (87)	77	Family #03	Neuromuscular	Unsteadiness + numbness (66)	Yes	Yes	Yes	NA	No
7	Norwegian	71	62	Family #04	Neuromuscular	Unsteadiness + numbness (40)	Yes	NA	NA	NA	Yes
8	British	Died (75)	71	Family #05	General neurology	Unsteadiness + dysarthria (70)	Yes	Yes	NA	NA	No
9	British	83	77	Family #06	Neuro-Otology	Unsteadiness (60)	Yes	Yes	Yes	Yes	No
10	British	80	70	Family #06	Neuro-Otology	Numbness (40)	Yes	Yes	Yes	No	No
11	British	78	74	Family #06	Neuro-Otology	Unsteadiness (70)	Yes	Yes	Yes	No	Yes
12	British	71	67	Family #07	Neuromuscular	Unsteadiness (40)	Yes	NA	NA	NA	Yes
13	British	68	64	Family #07	Neuromuscular	Positive sensory symptoms (50)	Yes	NA	Yes	No	Yes
14	British	74	73	Family #08	Neurogenetics	Unsteadiness (57)	Yes	Yes	Yes	Yes	No
15	British	69	64	Family #09	Neuromuscular	Unsteadiness (47)	Yes	Yes	Yes	Yes	No
16	British	66	57	Family #10	Neuromuscular	Numbness (55)	Yes	No	NA	NA	Yes
17	British	68	57	Family #10	Neuromuscular	Numbness (50)	Yes	NA	NA	NA	Yes
18	British	92	74	Family #11	Neuromuscular	Burning pain (47)	Yes	NA	NA	NA	No
19	British	95	79	Family #11	Neuromuscular	Unsteadiness + numbness (55)	Yes	NA	NA	NA	No
20	British	69	65	Family #12	Neuro-Otology	Unsteadiness (50)	Yes	Yes	Yes	No	Yes
21	British	69	51	Family #13	Neurogenetics	Unsteadiness (30)	Yes	No	NA	Yes	Yes
22	French	62	55	Family #14	Neuromuscular	Unsteadiness (51)	Yes	Yes	Yes	NA	Yes
23	French	73	68	Family #14	Neuromuscular	Unsteadiness (50)	Yes	Yes	Yes	NA	Yes
24	French	66	57	Family #14	Neuromuscular	Unsteadiness (55)	Yes	No	NA	Yes	Yes
25	French	68	64	Family #15	Neuromuscular	Unsteadiness (60)	Yes	Yes	Yes	NA	Yes
26	Italian	57	55	Family #16	Neuromuscular	Unsteadiness (52)	Yes	Yes	NA	Yes	No
27	Italian	68	64	Family #16	Neuromuscular	Unsteadiness (55)	Yes	Yes	Yes	Yes	No
28	Italian	66	61	Family #17	Neuromuscular	Positive sensory symptoms (47)	Yes	Yes	Yes	NA	Yes
29	New Zealander European	Died (59)	51	Family #18	General neurology	Unsteadiness (37)	Yes	Yes	Yes	Yes	Yes
30	New Zealander European	59	51	Family #18	General neurology	Unsteadiness + dysarthria (40)	Yes	Yes	Yes	Yes	No
31	New Zealander European	78	55	Family #19	Neurogenetics	Unsteadiness (55)	Yes	Yes	Yes	No	Yes
32	New Zealander European	54	47	Family #19	General neurology	Unsteadiness (45)	Yes	Yes	Yes	Yes	Yes
33	New Zealander European/ Māori	63	54	Family #20	General neurology	Numbness (41)	Yes	No	Yes	Yes	Yes
34	New Zealander European/ Māori	66	76	Family #20	Neurogenetics	Unsteadiness (19)	Yes	Yes	Yes	Yes	No
35	New Zealander European	Died (81)	74	Family #21	General neurology	Oscillopsia + postural hypotension (57)	Yes	Yes	Yes	Yes	No
36	New Zealander European	69	54	Family #21	General neurology	Unsteadiness (48)	Yes	Yes	Yes	Yes	No
37	New Zealander European	81	78	Family #22	General neurology	Numbness (73)	Yes	Yes	Yes	NA	No

ID	Nationality	Current age	Age at examination	Family history	Main clinic for follow-up	Symptom of neurological onset (age)	Sensory peripheral neuropathy	Cerebellar dysfunction	Bilateral vestibular areflexia	Dysautonomia	Cough
38	New Zealander European	75	72	Family #22	General neurology	Unsteadiness (70)	Yes	Yes	Yes	NA	No
39	New Zealander European	75	62	Family #23	General neurology	Unsteadiness (52)	Yes	Yes	Yes	Yes	No
40	Brazilian European	50	48	Family #24	Neurogenetics	Unsteadiness (43)	Yes	Yes	Yes	No	Yes
41	Brazilian European	53	53	Family #24	Neurogenetics	Oscillopsia (44)	Yes	Yes	Yes	No	Yes
42	Brazilian European	70	71	Family #25	Neurogenetics	Constipation (35)	Yes	Yes	Yes	NA	Yes
43	Brazilian European	64	65	Family #25	Neurogenetics	Constipation (40)	Yes	Yes	Yes	NA	Yes
44	British	74	60	Family #26	Neuromuscular	Unsteadiness (50)	Yes	Yes	Yes	NA	No
45	British	Died (78)	72	No	Neuromuscular	Unsteadiness (57)	Yes	Yes	NA	NA	No
46	British	49	37	No	Neurogenetics	Unsteadiness (30)	Yes	Yes	Yes	NA	Yes
47	British	73	54	No	Neuromuscular	Unsteadiness (54)	Yes	Yes	Yes	NA	No
48	British	67	55	No	Neurogenetics	Oscillopsia (50)	Yes	Yes	Yes	NA	No
49	British	76	64	No	Neuromuscular	Sexual dysfunction (50)	Yes	Yes	Yes	Yes	Yes
50	British	58	47	No	Neurogenetics	Unsteadiness + oscillopsia (45)	Yes	Yes	Yes	NA	No
51	British	80	74	No	Ataxia	Positive sensory symptoms (55)	Yes	Yes	NA	No	Yes
52	British	86	76	No	Neuromuscular	Unsteadiness + numbness (65)	Yes	Yes	NA	NA	No
53	British	Died (64)	54	No	Neuromuscular	Unsteadiness (45)	Yes	Yes	Yes	NA	Yes
54	British	61	51	No	Neuromuscular	Unsteadiness + positive sensory symptoms (50)	Yes	Yes	NA	Yes	No
55	British	80	69	No	Neurogenetics	Unsteadiness (65)	Yes	Yes	NA	NA	No
56	British	45	33	No	Neurogenetics	Unsteadiness + dysarthria (30)	Yes	Yes	NA	NA	No
57	British	60	52	No	Neuromuscular	Positive sensory symptoms (45)	Yes	NA	NA	NA	No
58	British	74	71	No	Neuromuscular	Unsteadiness (65)	Yes	Yes	Yes	NA	No
59	British	67	75	No	Ataxia	Unsteadiness (57)	NA	Yes	Yes	NA	Yes
60	British	77	72	No	Neuromuscular	Unsteadiness (63)	Yes	Yes	Yes	NA	No
61	British	Died (76)	62	No	Neuromuscular	Numbness (55)	Yes	No	NA	NA	Yes
62	British	75	72	No	Neuromuscular	Unsteadiness + numbness (67)	Yes	Yes	Yes	NA	No
63	British	Died (79)	67	No	Neurogenetics	Unsteadiness (57)	Yes	Yes	NA	NA	No
64	British	56	51	No	Neuromuscular	Unsteadiness (47)	Yes	No	NA	NA	Yes
65	British	62	55	No	Ataxia	Unsteadiness (50)	Yes	Yes	Yes	Yes	Yes
66	British	80	71	No	Neurogenetics	Unsteadiness (65)	Yes	Yes	Yes	No	No
67	British	71	67	No	Neuromuscular	Numbness (48)	Yes	Yes	Yes	No	Yes
68	British	77	65	No	Neurogenetics	Unsteadiness + numbness (55)	Yes	Yes	Yes	NA	Yes
69	British	81	74	No	Neuro-Otology	Unsteadiness (62)	Yes	Yes	Yes	NA	Yes
70	French	65	55	No	Neuromuscular	Unsteadiness + positive sensory symptoms (45)	Yes	Yes	Yes	NA	Yes
71	French	73	64	No	Neuromuscular	Unsteadiness (54)	Yes	NA	Yes	NA	Yes
72	French	75	69	No	Neuromuscular	Unsteadiness (58)	Yes	Yes	Yes	NA	Yes
73	French	60	55	No	Neuromuscular	Positive sensory symptoms (40)	Yes	No	No	Yes	Yes
74	French	78	58	No	Neuromuscular	Numbness (54)	Yes	Yes	NA	Yes	Yes
75	French	67	61	No	Neuromuscular	Unsteadiness + positive sensory symptoms (61)	Yes	No	NA	Yes	Yes

ID	Nationality	Current age	Age at examination	Family history	Main clinic for follow-up	Symptom of neurological onset (age)	Sensory peripheral neuropathy	Cerebellar dysfunction	Bilateral vestibular areflexia	Dysautonomia	Cough
76	French	51	46	No	Neuromuscular	Positive sensory symptoms (44)	Yes	No	No	NA	Yes
77	French	59	54	No	Neuromuscular	Positive sensory symptoms (50)	Yes	No	NA	NA	Yes
78	French	65	61	No	Neuromuscular	Positive sensory symptoms (55)	Yes	No	Yes	NA	Yes
79	French	68	65	No	Neuromuscular	Unsteadiness (47)	Yes	No	Yes	NA	Yes
80	French	72	70	No	Neuromuscular	Burning pain (20)	Yes	Yes	Yes	NA	Yes
81	French	61	59	No	Neuromuscular	Unsteadiness (50)	Yes	Yes	Yes	NA	Yes
82	Italian	66	62	No	Neuromuscular	Unsteadiness (56)	Yes	Yes	Yes	Yes	No
83	New Zealander European	75	67	No	General neurology	Postural hypotension (57)	Yes	Yes	Yes	Yes	No
84	New Zealander European	82	73	No	General neurology	Unsteadiness (72)	Yes	Yes	Yes	Yes	No
85	New Zealander European	Died (82)	67	No	General neurology	Unsteadiness (62)	Yes	Yes	Yes	No	No
86	New Zealander European	Died (72)	66	No	Neurogenetics	Unsteadiness (56)	Yes	Yes	Yes	Yes	No
87	Other European	73	67	No	General neurology	Unsteadiness (64)	Yes	Yes	Yes	Yes	No
88	British	70	68	No	Neurogenetics	Unsteadiness (56)	Yes	Yes	Yes	NA	Yes
89	British	78	78	No	Ataxia	Unsteadiness (60)	Yes	Yes	NA	NA	Yes
90	British	73	72	No	Ataxia	Unsteadiness (64)	Yes	Yes	NA	NA	No
91	British	79	71	No	Ataxia	Unsteadiness (50)	Yes	Yes	Yes	NA	Yes
92	British	72	69	No	Ataxia	Oscillopsia (62)	Yes	Yes	NA	NA	No
93	Slovenian	62	59	No	Neuromuscular	Unsteadiness (45)	Yes	Yes	Yes	Yes	Yes
94	Slovenian	64	62	No	Neuromuscular	Unsteadiness + Oscillopsia + dysarthria (52)	Yes	Yes	Yes	Yes	No
95	Slovenian	63	58	No	Neuromuscular	Unsteadiness (53)	Yes	No	Yes	Yes	No
96	British	Died (80)	57	No	Neurogenetics	Unsteadiness (57)	Yes	Yes	NA	NA	No
97	Italian	80	78	No	Neuro-Otology	Unsteadiness (76)	Yes	Yes	Yes	NA	Yes
98	Italian	62	56	No	General neurology	Unsteadiness (46)	Yes	Yes	Yes	NA	Yes
99	British	88	82	No	Neuromuscular	Numbness (60)	Yes	Yes	No	NA	Yes
100	British	87	76	No	General neurology	Unsteadiness + numbness (70)	Yes	Yes	NA	NA	No

NA: not available (subclinical involvement cannot be excluded since investigations were not performed)
